# Colorectal Cancer in Mississippi: How to Change the Tide of Bad News to a Success Story?

**DOI:** 10.7759/cureus.90164

**Published:** 2025-08-15

**Authors:** Sudheer Koutha, Srinivasan Vijayakumar, Catherine C Young, Felisa Wilson Simpson, Lilanta Joy Bradley

**Affiliations:** 1 Office of Health Surveillance, Research, and Evaluation/Office of Preventive Health, Mississippi State Department of Health, Jackson, USA; 2 Radiology, Ochsner Clinic Foundation, New Orleans, USA; 3 Radiotherapy and Oncology, Kasturba Medical College, Manipal, Manipal Academy of Higher Education, Manipal, IND; 4 Cancer Care, Cancer Care Advisors and Consultants LLC, Ridgeland, USA; 5 Mississippi Comprehensive Cancer Control Program/Office of Preventive Health, Mississippi State Department of Health, Jackson, USA; 6 Health Promotion and Chronic Disease Prevention/ Office of Preventive Health, Mississippi State Department of Health, Jackson, USA; 7 Community Medicine and Population Health, The University of Alabama, Tuscaloosa, USA

**Keywords:** cancer prevention, cancer screening, colorectal cancer, colorectal cancer screening, deep south, fecal occult blood test, mississippi, public health, screening colonoscopy

## Abstract

Colorectal cancer (CRC) remains one of the most preventable yet deadly cancers in the United States (MS), with Mississippi reporting the highest CRC incidence and mortality rates nationally. More importantly, this is largely attributed to late-stage diagnoses, which are more difficult and expensive to treat. Despite the proven effectiveness of CRC screening (CRCS) in early detection and prevention, screening uptake remains low in MS, particularly among socioeconomically disadvantaged and rural populations.

Public health challenges in MS are compounded by a high poverty rate, low educational attainment, high uninsured rates, and widespread provider shortages. Factors such as distance to basic healthcare services and underserved medical facilities create structural barriers that limit access to cancer prevention services, leading to delayed diagnoses and increased mortality. Additionally, MS has one of the lowest physician-to-population ratios in the United States, making it difficult to expand traditional provider-based screening programs.

To address these disparities, this review proposes a multi-level, precision population health strategy tailored to the state’s needs. The model integrates the Chronic Care Model (CCM), community health workers (CHWs), telehealth hubs, team-based care, and community education to expand CRCS access and effectiveness. CHWs, as trusted community liaisons, are uniquely positioned to bridge gaps in awareness, navigation, and follow-up. By training CHWs to support test selection, assist with test administration, and coordinate referrals and follow-up care, this model enhances outreach and engagement in underserved areas. Further, we recommend the application of key social determinants of health (SDH) indices such as the Area Deprivation Index (ADI), Social Deprivation Index (SDI), and Life Expectancy Index (LSI), to target high-risk populations. These composite indices help identify locations with the greatest need for intervention and resource allocation. When combined with emerging innovations in precision medicine, such as non-invasive tests and digital tracking tools, CRCS efforts can be made more efficient, scalable, and equitable.

In summary, this review outlines a comprehensive, data-driven strategy for improving CRCS uptake in MS, with broader implications for other Deep South states facing similar challenges. By delivering the right screening test to the right person at the right time, this approach not only supports early detection and reduces mortality but also contributes to long-term health disparity elimination and system-level transformation in cancer prevention and care.

## Introduction and background

Colorectal cancer (CRC) is a major cause of cancer-related mortality in the United States (US) and the state of Mississippi (MS). Furthermore, the incidence of CRC and mortality related to it in MS are increasing and unlikely to decrease unless immediate strategic measures are implemented. Fortunately, CRC is preventable in most cases. Primary, secondary, and tertiary prevention measures are applicable in CRC, and many such strategies have shown success [1]. For example, CRC screening (CRCS) with colonoscopy has been documented to decrease CRC-related mortality [2]. The use of CRCS strategies leads to the diagnosis of CRC at an earlier stage, thus making it easier to treat with improved survival rates and a better quality of life [3]. In addition, innovations and rapidly developing current strategies such as precision medicine [4] and precision population medicine [5] can play key roles in controlling CRC in MS. Lives can be saved, in addition to improving the QOL of those diagnosed with CRC.

The primary causes for the current CRC increase in MS include a lack of widespread CRC-related knowledge and information to the public in a format that uses state-of-the-art modern communication tools [6,7]. In addition, MS residents possess a high prevalence of risk factors such as obesity, diabetes, and unhealthy eating habits [8-13]. Lack of access to CRCS options, slow implementation of comprehensive statewide cancer care strategies, as well as the absence of National Cancer Institute-designated cancer center(s) in MS and neighboring states (Louisiana and Arkansas) of the Deep South, add to the adverse factors leading to a high incidence as well as high mortality rates related to CRC [14].

Our hypotheses to improve CRC outcomes in MS are the following: (a) The immediate cause of the CRC epidemic is due to a lack of sufficient CRCS; (b) The cause for the inadequate CRCS is a lack of sufficient knowledge and education about the importance of CRCS among the public. This, in turn, is related to a low high school and college-educated population. (c) The above two causations can be overcome in 5-10 years with the use of an aggressive educational campaign about the importance of CRCS with state-of-the-art information communication technology (ICT)-based interventions [15]. (d) A key to the success of such a rapid health education strategy is the presence of a robust community health worker (CHW) cadre in MS, who can play an active role in CRCS strategies [14]. A personalized approach to CRCS is essential to its success in MS, which has specific sociodemographic and educational profiles leading to poor social determinants of health (SDH), and many risk factors, and chronic health conditions as detailed above.

While a detailed discussion of the causes described above is beyond the scope of this review, it will focus on the (dismal) CRC and CRCS statistics in MS and new approaches to CRCS strategies that are likely to succeed. These proposed CRCS approaches may likely serve as a pilot idea to tackle other cancer care issues in MS [14]. An interdisciplinary approach is being proposed here with the inclusion of an oncologist, an epidemiologist, an experienced MS multidisciplinary healthcare expert and policy specialist, as well as a cancer care researcher familiar with the health issues of the Deep South, working together.

In the subsequent sections, we will review the following: (a) CRC statistics in MS as compared to that of other states in the Deep South, the US, and the world, (b) High-risk health data of MS relevant to CRC, (c) MS CRCS data, and its relationship to current CRCS strategies, (d) Effective CRCS strategies in the US and abroad, including recommendations for how they can be adapted to MS’s rural populations and demographics, (e) Importance of sociodemographic Index (SDI), Social Deprivation Index (SDI), Area of Deprivation Index (ADI), and Local Social Inequity (LSI) and their application to MS’s CRC epidemic, (f) Utilization of CHWs in MS as a multiplier force to enhance and expand future CRCS initiatives, (g) Current innovations in cancer biology, technology, and health sciences that can be applied to improve cancer care outcomes in MS with a specific focus on CRC and CRCS, and (h) Framework for policy decisions to assist MS in CRC control and overall cancer care management.

## Review

Accounting for 8% of cancer deaths worldwide, CRC is among the top three most diagnosed cancers [3,16]. Economic growth in developing countries is leading to a more sedentary life as well as consumption of more westernized diets with higher processed meat and fat, and increased caloric intake. Combined with longer life expectancy and a growing population, this change is leading to a significant rise in CRC cases [17]. In 2008, there were over 1.2 million new CRC cases and 608,700 deaths worldwide [18]. By 2022, cases had risen to nearly two million (ranking third among all cancers), and deaths had surpassed 903,859 (ranking second) (Table 1). This marks a 60% increase in new cases and a 50% increase in deaths over 15 years, with almost one million people dying from the disease in 2022 [19].

**Table 1:** Global incidence mortality rates for six most prevelant cancers in 2022 Source: GLOBOCAN 2022 [19]; Lincensed under CC BY-NC-ND 4.0, Attribution-NonCommercial-NoDerivatives 4.0 International

Cancer site	Incidence			Mortality		
	Rank	New cases	Percentage of all sites	Rank	Deaths	Percentage of all sites
Lung	1	2,480,301	12.4	1	1,817,172	18.7
Female breast	2	2,308,897	11.6	4	665,684	6.9
Colorectum	3	1,926,118	9.6	2	903,859	9.3
Prostate	4	1,466,680	7.3	8	396,792	4.1
Stomach	5	968,350	4.9	5	659,853	6.8
Liver	6	865,269	4.3	3	757,948	7.8

CRC rates are higher in the developed and industrialized nations; however, recently, CRC rates have increased in many developing countries as well [16].

**Figure 1:**
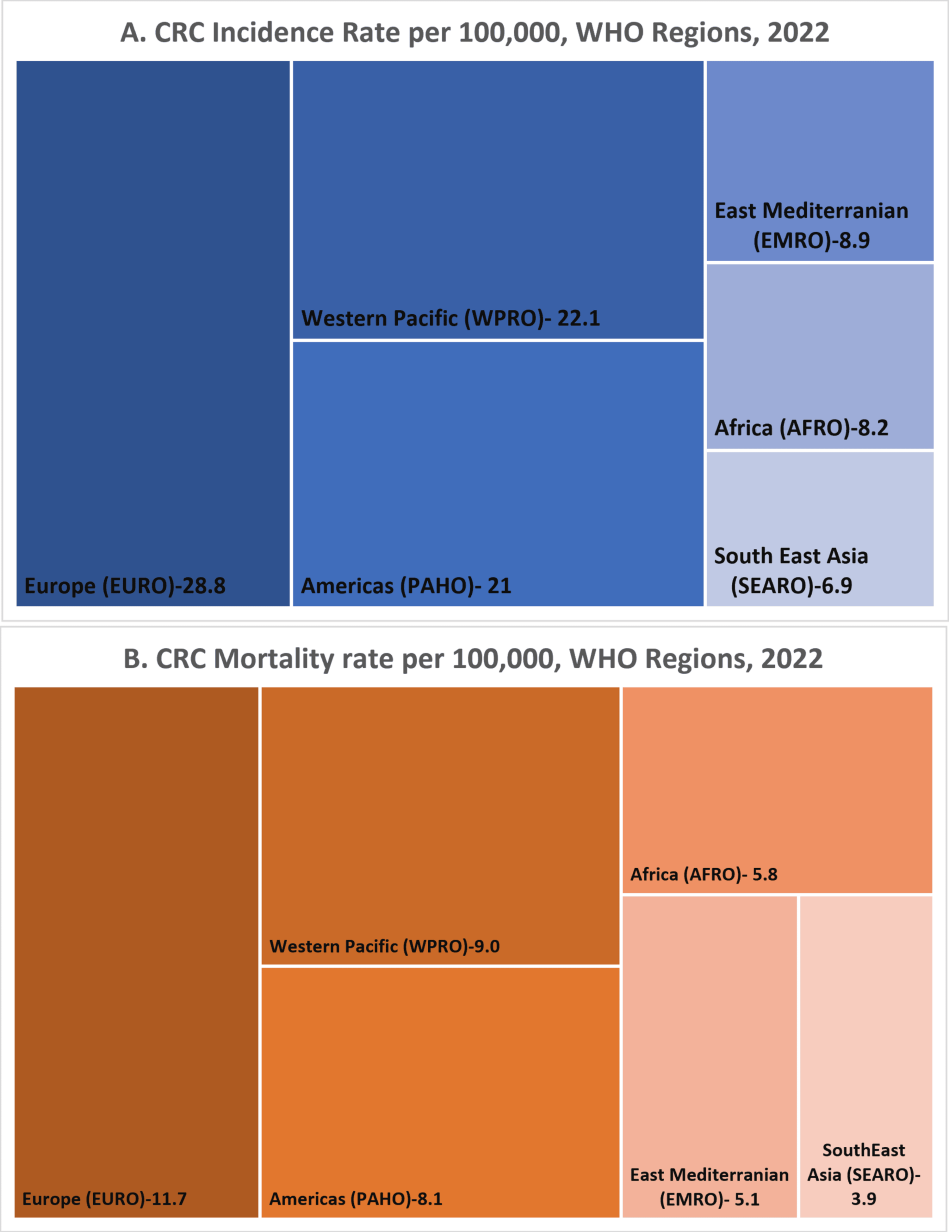
Comparison of colorectal cancer burden across WHO regions in 2022 CRC is most common in the European region (28.8 per 100,000), followed by the Western Paciﬁc (22.1 per 100,000) and the Americas (21.0 per 100,000). The lowest rates are in Southeast Asia (6.9 per 100,000) and Africa (8.2 per 100,000). Europe also has the highest death rate (11.7 per 100,000), while regions like Africa (5.8 per 100,000) and South-East Asia (3.9 per 100,000) show lower death rates, possibly due to underdiagnosis and reporting issues. Image Credit: International Agency for Research on Cancer, Cancer Today [21]

The World Health Organization (WHO) classifies member states into six regions to facilitate global health monitoring, reporting, and administrative coordination: the African Region (AFRO), Region of the Americas (RAHO), the South-East Asian Region (SEARO), the European Region (EURO), Eastern Mediterranean Region (EMRO), and Western Pacific Region (WPR). Each region encompasses countries with geographic, epidemiological, and cultural similarities, supporting tailored public health strategies and data comparisons across diverse global settings [20]. Figure 1 shows the age-standardized incidence and mortality rates, respectively, for CRC in the WHO regions in 2022 [21]. Figure 1 is interpreted with caution since the ability to collect high-quality data is not available in some developing nations. Therefore, the statistics must be considered incomplete.

Studies indicate several factors, such as drinking alcohol, smoking, eating red or processed meat, and having excess body fat, can increase the risk of CRC. Conversely, certain foods and habits like eating fiber-rich foods (fruits, vegetables, whole grains), consuming dairy, taking calcium supplements, and staying physically active can help lower the risk of CRC [22].

Remarkably, among developed countries, new cases of CRC are decreasing because more people are consuming healthier diets and are getting screened more regularly [23]. Modern CRCS efforts, including colonoscopies, which help find and remove abnormal pre-cancerous growth before it becomes malignant, are also important factors in this decline [24-26]. However, colonoscopies require substantial infrastructure, making them expensive in poorer regions worldwide, including in many disadvantaged regions within developed nations. A simpler and more affordable stool test, fecal immunochemical test (FIT), is a potential, first-step alternative for screening [27-30]. In an accompanying paper, the present authors describe various screening options and what strategies may help improve the screening rates in rural and healthcare-infrastructure-deficient regions like MS [Vijayakumar et al., Personal Communication]. Measuring the cancer care infrastructure is not easy. Various metrics do exist, and using these measures, the state of MS needs to improve [31,32].

CRC is becoming more common among younger adults (under 50 years of age) in developed nations as reported in Organization for Economic Cooperation and Development (OECD) countries, including the US, Canada, and Australia [33-40]. Experts believe this could be due to early-life exposure to risk factors like poor diet, lack of physical activity, and possible gut bacteria changes due to antibiotic use [41]. To address this trend, in 2018, the US Preventive Services Task Force (USPSTF) and other professional organizations lowered the CRCS recommended age from 50 to 45 years, ensuring more ‘at-risk’ people are tested earlier. This can lead to earlier detection (at earlier stages) with better chances of successful treatment [42,43]. By 2040, the number of new CRC cases is expected to reach 3.2 million, with 1.6 million deaths worldwide [44]. Most of these cases are likely to happen in countries with high or very high Human Development Index (HDI) levels, mainly due to poor lifestyle habits.

CRC burden in the US

**Figure 2:**
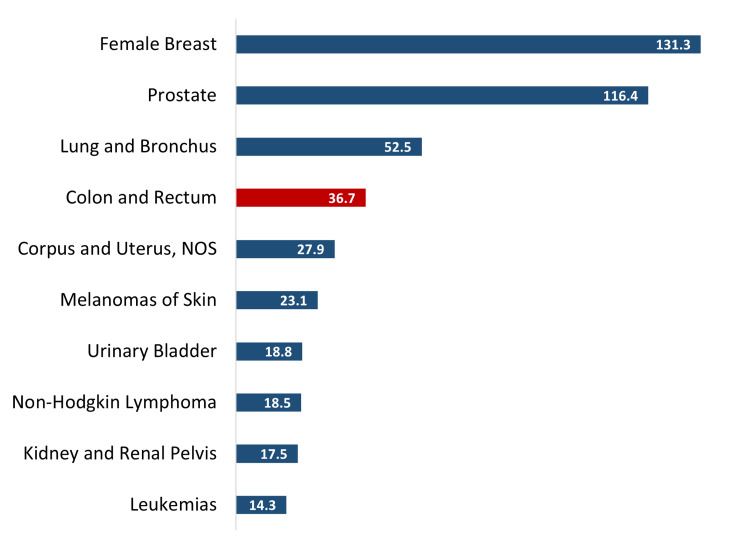
Top 10 cancers by rates of new cases per 100,000 in the United States, 2018-2022 Image Credit: Authors; Data Source: United States Cancer Statistics, CDC [45]

Figure 2 shows that CRC is the fourth common cancer among newly diagnosed cases (Incidence rate: 36.7 per 100,000) affecting more than 700,000 Americans during 2018-2022 in the US. More than 261,620 deaths occurred in the US from 2018 to 2022, with an adjusted mortality rate of 12.8 per 100,000 [42]. The National Cancer Institute estimates that 1,392,445 people are living with CRC in the US [46]. Considering the above, a complex picture of CRC incidence and prevalence emerges. Due to previously prevalent poor nutrition and other daily living habits, CRC incidence was increasing in OECD regions. However, with increased public education, knowledge, and awareness of the importance of diet and nutrition and CRCS in decreasing CRC incidence in Western societies, the incidence of CRC is starting to decline. On the other hand, regions with poorer socioeconomic and educational metrics, even within developed nations, are not experiencing a similar decline. Thus, the populations in these resource-scarce areas have higher incidences and mortalities of CRC and lower CRCS rates. The state of MS is a good example of such a region. The lack of CRC knowledge, health education, and promotion of CRCS, as well as the lack of CRCS methods such as colonoscopies, is leading to an increase in the incidence of CRC in these countries and states. In this context, MS can be considered as a ‘microcosm’ of the Global South, and the innovations that succeed in MS may also help the Global South.

Sociodemographic metrics of Deep South versus comparable ‘Northern’ states

According to the New World Encyclopedia, “the Deep South is a descriptive category of cultural and geographic subregions, the belt stretching from the Atlantic Ocean to west of the Mississippi River primarily consisting of five states, South Carolina, Georgia, Alabama, Mississippi, and Louisiana, although a sixth state, Arkansas, is often included [47]. The evolution of the Deep South in the US involves a complex history encompassing various aspects such as politics, economy, and ancestral origins [48-50]. Detailed descriptions of these elements are beyond the scope of this paper. Those elements influencing the evolution of the Deep South have also influenced the health status of its population. In Table 2, a few important sociodemographic and economic metrics of the six states of the DS are compared with the national metrics of the US, as well as four selected Midwest and Northeastern states.

**Table 2:** Sociodemographic and economic metrics of the Deep South versus the entire country as well as four ‘comparable northern’ states Sources: [51-58]; *For college graduation percentages, statistics are for the share of people aged 25 and older with a bachelor’s degree. Mississippi (MS) state had the least per capita GDP in 2023. Maine (ME) and Vermont (VT) are included because their sizes in square miles are lower than the Deep South states yet have a higher percentage of rural population. They also have higher education percentages and lower percentages of uninsured population. Ohio (OH) and Pennsylvania (PA) are included because their areas fall between the largest and smallest Deep South states – between Georgia (GA) and South Carolina (SC). GDP: Gross Domestic Product

Region/state	Per Capita GDP for 2023 (in constant 2017 US dollars)	Percentage of Graduation		Percentage of Home Ownership (2024)	Percentage of (Healthcare) Uninsured Population, 2023	Percentage of Rural Geography	Geographic Area (Sq. miles)
		High School %, 2025	College* %, 2024				
United States	66,813 (82,769 In 2023 $)	89.4	36	65.9	8.0	20.4	3,796,677
MS	39,102	86.6	26	75.5	10.3	54.4	48,432
LA	52,078	86.9	27	67.3	6.9	29.1	52,378
GA	59,942	89.0	35	65.5	11.4	26.6	59,424
SC	48,372	89.6	33	73.0	9.1	31.8	32,020
AL	47,324	88.1	29	73.8	8.5	42.0	52,420
TN	59,694	89.6	32	68.9	9.3	34.7	42,144
ME	52,862	94.5	37	75.5	5.9	61.5	35,380
VT	54,170	94.5	44	73.7	3.4	66.1	9,616
PA	60,910	91.9	35	71.0	5.4	23.6	46,054
OH	59,241	91.6	32	66.6	6.1	24.0	44,826
AR	45,892	88.6	26	65.9	65.9	43.9	53,179

In 2023, MS had the lowest per capita GDP among the Deep South states and ranked among the lowest in the US's most rural states in key socioeconomic indicators. In contrast, northeastern rural states like Vermont (VT) and Maine (ME) reported lower percentages of uninsured individuals and higher rates of high school and college graduates compared to the Deep South [54]. These factors seem to contribute to better health outcomes, including higher CRCS rates and lower incidence and mortality from CRC in these states (Table 3). VT reported a CRC incidence rate of 32.7 and a mortality rate of 12.8 per 100,000, while ME had an incidence rate of 35.0 and a mortality rate of 12.6 per 100,000. Notably, ME ranked third in the nation for CRCS rates. The states of AL, AR, LA, and MS had significantly fewer college graduates compared to other states and the national average, which may lead to unfavorable CRC outcomes in the DS states. Despite socioeconomic challenges, MS has the highest homeownership rate (75.5%) among the states compared, which may reflect community stability and a potential asset for public health outreach. However, MS faces other significant health disparities. These comparisons shown in Table 2 underscore the urgent need to strengthen and expand CRCS efforts in the Deep South, particularly in MS, to address preventable cancer burden and improve health equity across rural communities.

**Table 3:** Variation of colorectal cancer outcomes and screening prevalence in Deep South states in the United States Maine (ME) and Vermont (VT) are included in this table and Table 3 because their sizes in square miles are lower than the DS states yet have a higher percentage of rural population. They also have higher education percentages and lower percentages of uninsured population. Ohio (OH) and Pennsylvania (PA) are included because their areas fall between the largest and smallest DS states – between Georgia (GA) and South Carolina (SC).

State/Area Name	Incidence	Mortality	Screening %	5-year Survival Rate	Late-stage Incidence Rate
MS	46.4	17.6	62.5	59.7	29.0
LA	44.5	15.2	69.3	64.0	25.8
AR	41.1	15.0	64.0	64.0	25.1
TN	38.7	14.7	64.3	63.1	23.8
AL	40.1	14.4	67.7	62.7	22.1
GA	39.4	13.8	66.0	63.2	22.9
ME	35.0	12.6	72.2	67.2	20.5
VT	32.7	12.8	69.6	69.3	18.6
OH	38.9	13.9	67.6	64.8	23.9
PA	37.2	13.1	66.9	64.1	23.4
US	36.4	12.9	66.9	64.4	21.8

Comparison of CRC burden among Deep South states versus four ‘comparable Northern’ states and the US

At least five of the six Deep South states had incidence rates 10% higher than the national average, and four of them had mortality rates 10% higher than the national average, as shown in Table 3. The screening prevalence and five-year survival rate in MS were significantly lower compared to the other Deep South states. In MS, about 62.5% of the cases are diagnosed at advanced stages, which was 85% higher than the national average (33.8%), and similarly worse than the four ‘northern’ states compared in Table 3 [59].

Current ‘bad news’ in Mississippi related to CRC and CRCS

MS is a mostly rural state. The state struggles with high poverty and unemployment [60]. In 2023, nearly one in five people lived below the poverty line [61]. The average household income in MS is $49,111, much lower than the national average of $70,784 [62]. About 15% of adults in MS do not have a high school diploma (Table 2), and nearly one in 10 adults does not have any health insurance coverage [63]. Most counties in MS do not meet the travel time and distance guidelines set by the Health Resources and Services Administration. In the MS delta, some people must travel approximately 75 miles to reach basic healthcare services [64]. All 82 counties in MS are considered medically underserved because of a lack of healthcare providers and facilities [65].

**Figure 3:**
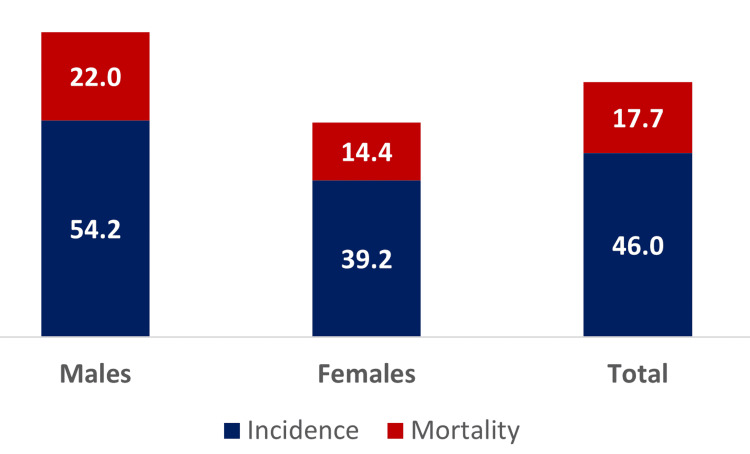
CRC incidence and mortality per 100,000 by sex in Mississippi (2018-2022) Image Credit: Authors [68]

MS reported the highest CRC mortality rate in the nation, with an age-adjusted rate that was 35% higher than the national rate (17.6 versus 12.9 per 100,000 people) during 2018-2022 [66]. The state reported the highest CRC incidence rate in the nation, with an age-adjusted rate that was 27.5% higher than the national rate (46.4 versus 36.4 per 100,000 people) during 2017-2021 [67]. It is estimated that in MS, an average of 1,700 new cases of CRC are diagnosed each year, and 630 people die from it. Male individuals (22.0 per 100,000) in MS had a 53% higher mortality rate when compared to female individuals (14.4 per 100,000) during 2018-2022, and only 38.2% higher incidence rate (Male: 54.2 per 100,000, Female: 39.2 per 100,000) (Figure 3). The rate of cases diagnosed with the early stage of CRC was 16.3 compared to the late-stage rate of 28.9 during 2018-2022 per 100,000 [68]. These numbers indicate the need for better and more effective, and successful CRCS strategies in the state.

Importance of CRCS

Without effective, affordable interventions and innovative strategies to increase CRCS, by 2040, the global burden of colorectal cancer will increase to 3.2 million new cases per year (an increase of 63%) and 1.6 million deaths per year (an increase of 73%) [44]. Fortunately, CRC is highly treatable when detected early using cost-effective screening tests and is often preventable when precancerous changes are detected and treated quickly. The CRC cases identified in the early stages have a remarkable five-year survival rate of 92.0% [69]. CRCS could detect asymptomatic cancer and ensure a better prognosis by diagnosing at earlier stages. It could also prevent the development of CRC by detecting precancerous polyps for removal [70].

For every 100,000 US residents eligible for screening, a 10-percentage point increase in the use of cancer screening strategies recommended by the USPSTF was estimated to prevent 226 deaths from lung cancer, 283 deaths from CRC, 82 deaths from breast cancer, and 81 deaths from cervical cancer over the lifetime [70]. Given that CRCS is associated with the highest number of preventable deaths among the cancers listed, this underscores its exceptional impact, making it one of the most powerful tools in cancer prevention and a critical public health priority.

Poor screening rates in Mississippi and their implications

**Figure 4:**
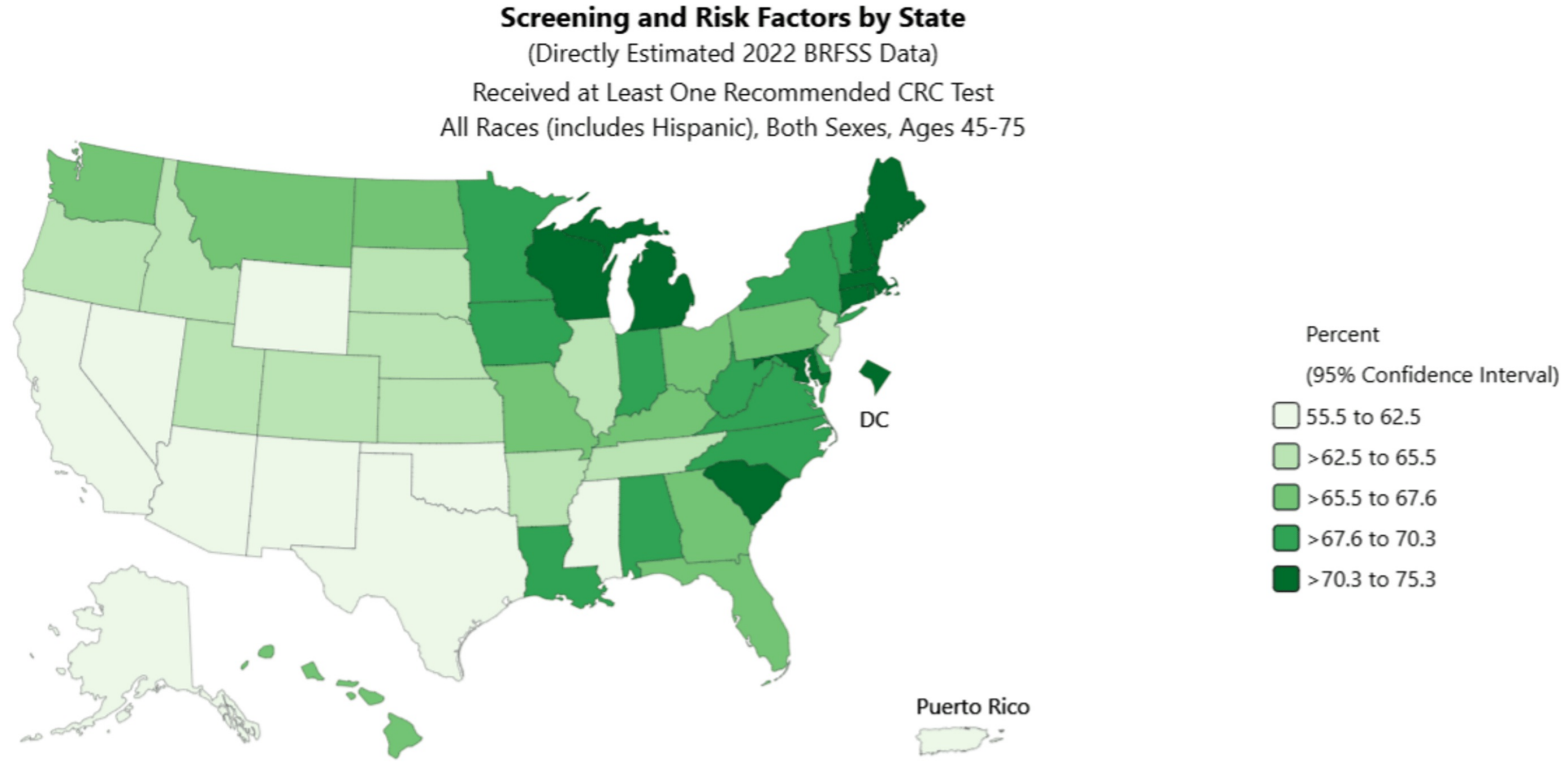
Colorectal cancer screening in the United States at state level (percentage of individuals who received at least one recommended colorectal test in 2022) Image Credit: National Cancer Institute [71]; Data Source: 2022 BRFSS Survey Data is the source for this data collected by the Behavioral Risk Factor Surveillance System (BRFSS) sponsored by the Centers for Disease Control and Prevention. Data for the United States is a median and not a percent. BRFSS Prevalence estimates presented here may vary from other published estimates due to differences in the methodology used to generate estimates; Data for United States does not include Puerto Rico

Studies show that early screening and detection help lower deaths from CRC. In the US, about 66.9% of adults have had at least one of the recommended screening tests performed. More women (68.2%) get screened for CRC in the US compared to men (65.6%). Figure 4 shows CRCS rates across the United States. Those eligible received at least one recommended CRC test (in the year 2022) that led to their inclusion in the analysis shown in Figure 4 [71].

**Figure 5:**
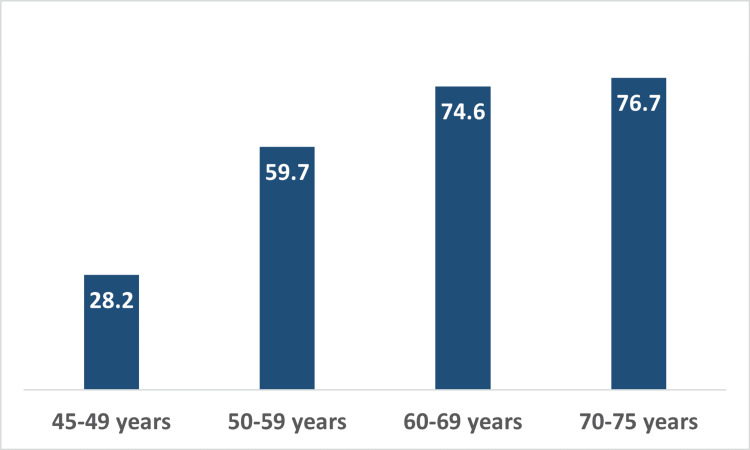
Colorectal cancer screening (%) by age group in Mississippi (2022) Data presented were obtained directly from the Mississippi BRFSS data set and represent 2022 BRFSS data specific to Mississippi. These numbers are generated internally by epidemiologists at Mississippi State Department of Health using SAS software.

MS has one of the lowest CRCS rates in the US among adults aged 45-75 years. In 2022, only 62.5% of people in this age group completed at least one recommended screening test within the suggested time frame [63]. Screening was lower in MS among men (60%) compared to women (65%). The percentage of people getting screened for CRC increases with age, but screening rates remain lowest among those aged 45-49 (Figure 5). This highlights a critical gap in the early detection of CRC. Strengthening screening efforts for adults aged 45-60 is likely to improve outcomes such as decreasing CRC-related mortality in MS. CRCS in this age group can prevent many deaths, reduce the need for expensive treatment, and help people continue to be productive during key working years. Additionally, lowering healthcare costs would improve the state’s economy by increasing workforce participation and economic contributions.

Quantitative measurement of socioeconomic gradations and their relationship with CRC issues

The SDH framework has been a global approach to investigating the drivers of health outcomes. Over the years, researchers have sharpened their understanding of how to access and measure key causal factors affecting health status. Among these efforts are SDH indices that are area-level measures used to assess the socioeconomic status of populations. Table 4 outlines the purpose, indicators, scale, and reference range, and creators of four indices that can be used to better understand the population-level impact of SDH on CRC outcomes.

**Table 4:** Description of SDH composite measures to explore colorectal cancer outcomes SDH: social determinants of health; SES: socioeconimic status; HRSA: Health Resources and Services Administration

Measure	Purpose	Indicators	Reference Range	Source/Institution of Origin	Year first introduced in the public domain and reference
Social Vulnerability Index (SoVI)	Aims to identify vulnerable communities most likely requiring support for natural disasters, hazardous events, and disease outbreaks	Sixteen social factors within four themes: SES, household characteristics, racial and minority status, and housing type & transportation	0 to 1 (the higher numbers indicate a higher level of vulnerability)	CDC/ University of South Carolina	2010 [72]
Social Deprivation Index (SDI)	Used to understand extent of social disadvantage in a community	Seven key domains: poverty, education, household composition, housing characteristics, access to a vehicle, unemployment	1 to 100 (higher numbers indicate higher disadvantage level)	Graham Center	2012 [73]
Area of Deprivation Index (ADI)	Used to improve access and facilitate a broad application of neighborhood-disadvantage metric inclusive of all US neighborhoods	Four key domains: income, education, employment, housing quality across 17 variables	1 to 100 (higher numbers indicate higher disadvantage level)	HRSA/ University of Wisconsin-Madison	2019 [74]
Local Social Inequity (LSI)	Provides localized life expectancy risk scores constructed with a health equity focus	over 200 area-level variables across ten domains	Q1 to Q5 (the higher quartile indicates the highest lower life expectancy risk)	RTI Health Solutions	2023 [75]

The overarching goal of these composite measures is to find a comprehensive way to assess which SDH factors’ influence can determine more efficacious therapeutics, community health interventions, policy making, and informed research for CRC prevention, treatment, and survivorship [76]. Key indices in the SDH framework include the ADI and the SDI. ADI ranks neighborhoods based on socioeconomic disadvantage [74]. SDI is a newer tool used to quantify the socioeconomic variation in health outcomes. Fully embracing the drivers of SDH as the underlying mechanism of fundamental issues impacting health outcomes continues to be a challenge for health researchers and practitioners. Until issues such as neighborhood disadvantage receive greater attention and measures such as SoVI, SDI, ADI, and LSI are prioritized (Table 4), we will continue to experience widening gaps in health disparities in CRC outcomes. Where one lives, works, and plays matters. There is a symbiotic relationship between one’s socioeconomic status and their neighborhood. However, some researchers have found that there are differences in health outcomes when comparing poor people who live in wealthier neighborhoods and poor people who live in disadvantaged neighborhoods [77,78]. This is not new information, but what is new is the shift in measuring poverty as an individual problem to measuring poverty as a system-level problem. For instance, several studies reported poverty rates in neighborhoods as opposed to individualistic poverty measures to show disparities in CRC survival across geographic areas [79-83].

Among the four SDH composite indices detailed in Table 4, the ADI provides a more focused assessment of socioeconomic status in a neighborhood, while SoVI and SDI offer broader perspectives on social deprivation by incorporating a wider range of social and demographic characteristics. LSI is the newest composite measure that uses a more nuanced lens to capture over 70% of neighborhood variation in life expectancy across the US, as compared to ADI and SDI, which only account for 29-34% of the life expectancy variation nationally [84]. Applying the appropriate indices to CRC populations requires careful consideration in clinical research and practice based on intended use and variables’ relevance. Even with the myriad applications of these indices on chronic disease outcomes already established, unfortunately, there is a dearth of studies that have demonstrated their use and relevance in CRC incidence, CRCS utilization, and CRC outcomes specifically.

A study by Durfey et al., published in 2019, described the impact of neighborhood disadvantages on chronic disease management, highlighting that individuals in socioeconomically disadvantaged areas often face barriers to effective chronic disease management, including limited access to healthcare resources and support systems [85]. In 2022, Sadler et al. highlighted a geospatial healthfulness index, demonstrating its utility in identifying areas with higher chronic disease prevalence and poorer health outcomes [86]. This index can be instrumental in targeting interventions and resources to communities that are at higher risk for chronic disease. In 2023, Lee et al.'s study associated county-level social vulnerability with chronic respiratory disease mortality and found that counties with higher SoVI experienced greater mortality rates from chronic respiratory diseases, underscoring the importance of addressing social determinants in chronic disease management [87]. Collectively, these studies emphasize the critical role of indices such as neighborhood disadvantage, healthfulness, and social vulnerability in informing public health strategies. By leveraging these tools, public health initiatives can be more effectively tailored to address the specific challenges faced by rural populations in managing chronic diseases, especially in Deep South states.

The strength of the CHW program in Mississippi and its potential application in CRCS

CHWs can play a vital role in cancer prevention and improving cancer outcomes in many communities that are marginalized and lack equitable screening options. As a trusted member of their communities, CHWs can play an important role in educating, linking people to services, and providing follow-up care for CRC initiatives. Unlike a traditional provider, CHWs typically reside and have a vested interest in the communities they serve. With the increasing number of physician shortages [88], CHWs are uniquely positioned to bridge the gap between communities and the health care system. The Association of American Medical Colleges (AAMC) predicted that by 2030, the demand for doctors will outstrip the supply and that the US will experience a shortage of up to 121,300 physicians [88]. MS is the only state expected to have an F grade in physician availability, with 118 active physicians per 100,000 people, which is 42% below the national mean of 203 [89]. There is growing evidence that shows integrating CHWs into clinical settings using health informatics-based strategies can help provide coordinated patient care and foster health-promoting behaviors [90]. Community-clinical linkage models provide opportunities to enhance patients’ awareness and self-efficacy related to disease prevention and management and have the potential to improve quality of care and patient outcomes, resulting in decreased health disparities among minority and other socioeconomically disadvantaged populations, especially in the context of chronic disease.

Evidence-based studies have been conducted documenting investigative findings to support the use of CHWs in clinical-community linkages, which can improve cancer outcomes, showing the effectiveness of CHW/patient navigator programs in ultimately increasing the rate and improving the timeliness of breast, cervical, and CRCS in federally qualified health centers (FQHCs) [91]. According to Roland et al., use of CHWs and patient navigators in FQHCs further supports the effectiveness of programs utilizing CHW/patient navigator mode of intervention to increase cancer screening, most often through promoting breast, cervical, and colorectal cancer screenings and/or referral for diagnostic evaluation [92]. In addition to the effectiveness and efficiency of the use of CHWs to bridge the gap in cancer screening uptake, it has also been widely demonstrated to be cost-effective. The Community Preventive Services Task Force (CPSTF) recommended the engagement of CHWs to increase cancer screening from an economic lens based on a systematic literature search [93]. Findings revealed that the engagement of CHWs to increase cervical and CRC screenings is cost-effective when the estimated cost-effectiveness ratios were found to be less than a conservative $50,000 per quality-adjusted life year threshold. Further, the authors found that quality-adjusted life years saved as a result of CRCS via colonoscopy were related and yielded net healthcare cost savings. This model or similar models can be applied in MS and other Southern states, including Deep South states that are predominantly rural and lack access to social service referrals (Tables 2-4).

Applying the continuum care model (CCM), CHWs, telehealth, smartphone apps, team-based care, and community education to improve CRCS in MS

To address the CRC epidemic in MS and/or the Deep South and its devastating human cost in mortality, morbidity, and loss of QOL, as well as societal cost of lost productivity, increased health care expenses, human suffering, and family breakups, several factors can be considered. These include adopting an advanced CCM, creating a telehealth hub, integrating CHWs into team-based care, standing orders for screening tests, use of advanced technology, and targeted awareness campaigns [14]. CCM is a concept involving an integrated system of care that guides and tracks patients over time through a comprehensive array of health services spanning all levels of intensity of care. Too many Mississippi residents are dying from late-stage diagnosis of one of the most preventable cancers due to many factors discussed earlier, especially the poor CRCS strategies and public health education. Incorporating an innovative CCM can address tracking a patient using advanced technology from an initial encounter with a provider using telehealth to screening and follow-up to help encourage patients who are not adherent, for example, those who do not return for a second level of testing with colonoscopy after a take-home abnormal screening test, such as FIT. This can also include incorporating CHWs as part of the CoC team model to address social drivers to access care and follow-up. There is evidence that shows telehealth can be cost-effective, useful, and can help with rural access in states like MS. In recent years, telehealth has been rapidly developed to realize more efficient chronic health management [94]. Telehealth is a way to provide healthcare remotely, facilitated by mobile technology like smartphone apps and the internet. Since MS has a substantial rural population, the use of telemedicine and mobile technology concepts is likely to improve CRCS and increase the percentage of CRC patients being diagnosed at an earlier stage. These innovations can also detect and treat precancerous colorectal lesions and polyps, and thus prevent further development to CRC, and thus improve survival outcomes [14].

Creating innovative CRCS programs for Mississippi: a new step forward

Many of the principles enunciated regarding PPM’s role in cancer prevention apply to CRCS [4]. Previous reports [4,5] emphasized the importance of ‘using the right health care intervention at the right time for the right population’. These ideas include applying state-of-the-art innovations such as genomic medicine, big data, electronic health records, artificial intelligence, wearable devices, and new ways of conducting clinical trials. Furthermore, the significance of interdisciplinary team approaches in addressing contemporary healthcare challenges was emphasized by Singh et al. [95]. Only such approaches can help improve the CRCS and CRC outcomes in MS.

**Figure 6:**
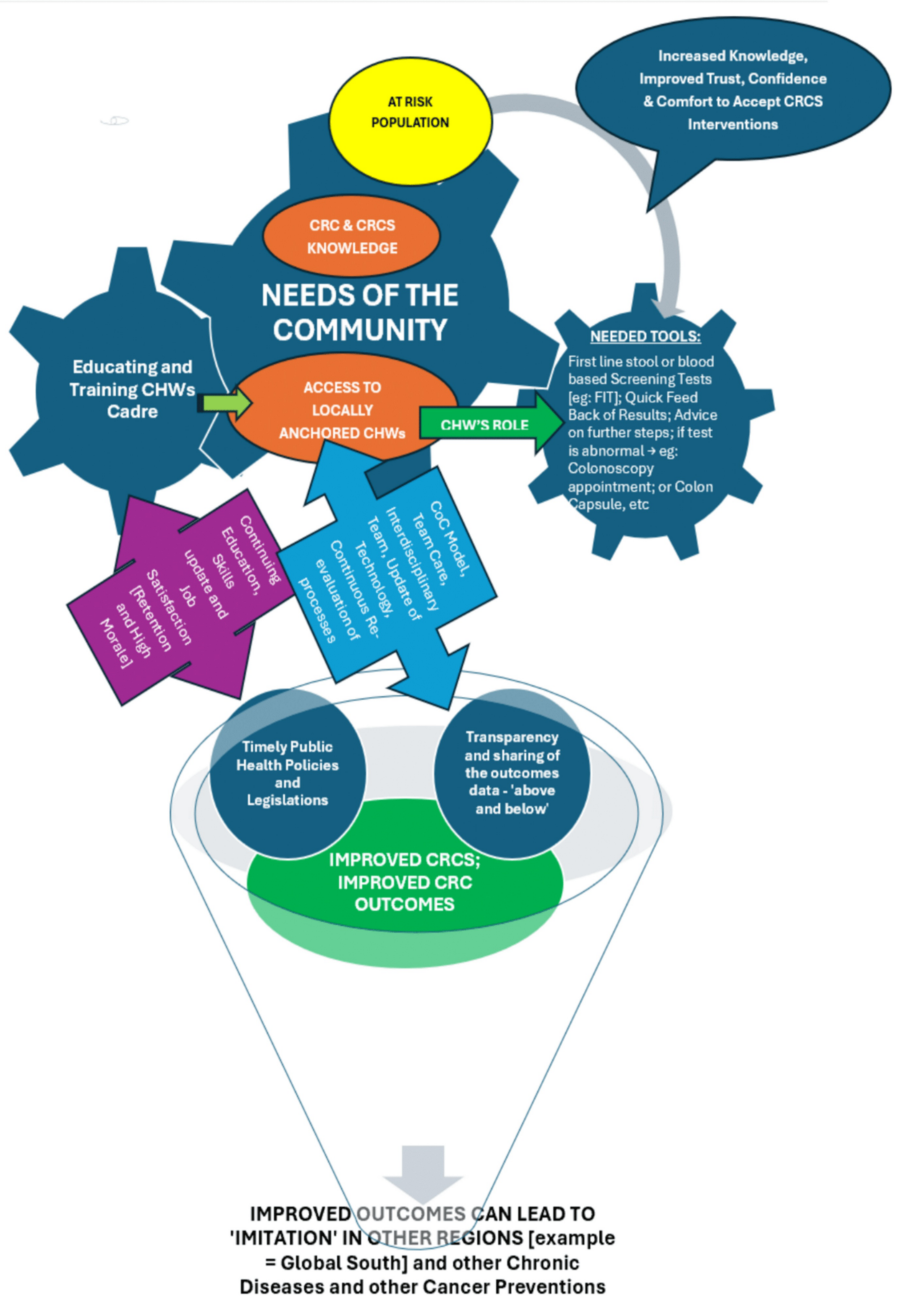
Role of continuum care model, community health workers, telehealth, smart-phone apps, team-based care, and community education to improve colorectal screening in Mississippi Image Credit: Authors

Based on the complex set of circumstances that led to increasing CRC in the state of MS (and in the neighboring states of the Deep South), detailed earlier, an interdisciplinary plan of action is proposed in Figure 6. The plan uses the CCM to educate residents of MS about CRC risks and the importance of CRCS in preventing unnecessary morbidity and mortality from CRC. Education efforts would be spearheaded by CHWs, who in turn would be provided with adequate knowledge, skills, and tools by a professional team of oncologists, surgeons, epidemiologists, healthcare policy professionals, and healthcare educators. CHWs, who are usually anchored to the local community, would not only educate at-risk populations about CRC and CRCS but also help them with choosing the ‘right option of CRCS tests that suits the circumstances and choices of everyone eligible for CRCS. CHWs would serve as key members of the team who advise the individual at risk about CRCS’s benefits and contraindications, advantages, and disadvantages of various tests, inform those who undergo a CRCS test of the results of the given test, and provide further recommendations based on the test results. CHWs would also facilitate access to further tests, if necessary, for example, for those whose fecal occult blood test showed an abnormal result and need a colonoscopy. After a colonoscopy, CHWs would help the individual understand the findings and work with the healthcare team to further advise on follow-up tests at appropriate times.

The strength of the existing CHWs program in MS can be leveraged to rapidly develop a comprehensive CRCS strategy that can incorporate the ‘right CRCS test for the right at-risk person at the right time’, an essential principle of PPM. Given the rural nature of the state of MS and its limited infrastructure resources, the following principles should be acceptable to the MS residents and their care providers: (a) In the immediate term (the next two to three years), FIT or one of the Cologuard® tests can be offered as a choice of the first line testing. Most studies show that a non-invasive fecal or blood test is preferred instead of a direct choice of colonoscopy by most ‘average risk’ individuals for CRC [96,1]. (b) If occult fecal blood was found, the options of fibro-optic sigmoidoscopy or a colonoscopy would be presented to the person at risk, and his/her choice would be respected. Other options, such as CT colonography, would also be presented using lay terms. (c) The CHWs would closely collaborate with the CRCS care providers to facilitate scheduling, performance, and follow-ups.

## Conclusions

CRC in the state of MS and the Deep South is a major cause of cancer-related morbidity and mortality that is highly preventable. With the progress in CRCS options, CRC incidence and related mortality are declining in most regions of Western nations. With an interdisciplinary approach to find some immediate and long-term solutions to the CRC ‘epidemic’ in MS and the Deep South, this review article proposes some innovative new approaches that are grounded in using already existent strengths of resources in MS, such as the CHWs program. The solutions proposed here can yield relatively rapid results in the immediate future, three to five years. In addition, leveraging some of the new concepts of PPM, substantial progress can be made in the intermediate and long-term as well. Of course, to achieve these optimistic improvements in outcomes in CRC, there is a need for a government-industry partnership to be developed rather quickly. Such new models and approaches proposed in this article can further help improve outcomes in other cancers and other chronic diseases as well in MS and the Deep South. Such paradigm shifts in public health planning in CRC and CRCS for MS, the Deep South, and the US can, in fact, serve as a pilot demonstration project for the Global South as well in these periods of ‘thinning’ healthcare resources globally.
